# The Value of an Ecological Approach to Improve the Precision of Nutritional Assessment: Addressing Contributors and Implications of the “Multiple Burdens of Malnutrition”

**DOI:** 10.3390/nu16030421

**Published:** 2024-01-31

**Authors:** Daniel J. Raiten, Alison L. Steiber, Omar Dary, Andrew A. Bremer

**Affiliations:** 1 Pediatric Growth and Nutrition Branch, Eunice Kennedy Shriver National Institute of Child Health and Human Development, National Institutes of Health, Bethesda, MD 20817, USA; andrew.bremer@nih.gov; 2 Academy of Nutrition and Dietetics, Chicago, IL 60606, USA; asteiber@eatright.org; 3 USAID, Bureau for Global Health, Division of Nutrition and Environmental Health, Washington, DC 20523, USA; odary@usaid.gov

**Keywords:** diet, nutrition ecology, food insecurity, malnutrition, nutritional assessment

## Abstract

Globally, children are exposed to multiple health risks associated with diet and nutrition. Rather than simply being a condition of having too much or too little food, malnutrition is more a syndrome comprising multiple burdens of coexisting and reciprocal malnutrition, infection, or other conditions. Importantly, children with such syndromes (e.g., stunting and anemia, which are neither specific nor necessarily sensitive to nutritional status) are more likely to also have irreversible functional outcomes such as poor growth, impaired immune function, or cognitive delays. The global health community has identified nutrition-related targets (e.g., Sustainable Development Goals (SDGs) and World Health Organization (WHO) Global Nutrition Targets) that, for multiple reasons, are difficult to address. Moreover, as the complexity of the global health context increases with persistent pandemics of infectious diseases and the rising prevalence of noncommunicable diseases, there is a growing appreciation that conditions selected as nutrition/health targets indeed represent syndromes for which nutritional status serves as both an input and outcome. In recognition of the impact of these combined challenges and the role of the multiple manifestations of malnutrition, we suggest an approach to nutritional assessment that is intended to improve the precision of context-specific, equitable approaches to health promotion, disease prevention, and treatment.

## 1. Introduction

Globally, a holistic public health systems approach to both nutrition and food security has focused primarily on ensuring the adequate intake of macro- and micronutrients. Examples of global health targets with presumed linkages to either macro- or micronutrient deficiencies include, but are not restricted to, anemia, wasting and underweight, and long-term manifestations, such as stunting. These consequences, but in particular anemia and stunting, represent multifactorial conditions that demand an approach that is inclusive and not limited to simplistic “too much or too little” supply-oriented paradigms [1]. Moreover, as highlighted by the report of *The Lancet Commission* [2], we are increasingly confronted by a “synergy of pandemics” (i.e., a global syndemic of malnutrition and climate change), which is creating increasing challenges for the global public health enterprise. As with other global targets, this syndemic is generally and understandably presented in the context of policy and programmatic challenges. However, to move the field forward, we need to improve the precision of assessment, attribution, and interventions to better address the clinical (health and developmental) impact of global challenges like anemia and stunting (“multiple burdens of malnutrition”) within the context of a changing global environment (including the impact of climate change).

Our progress in addressing these challenges to date continues to be slow. There has been insufficient progress to reach either the 2025 World Health Assembly (WHA) global nutrition targets or Sustainable Development Goals (SDGs). Regrettably, as highlighted in the recent UNICEF–WHO–The World Bank joint report [3], only about one-third of all countries are currently “on track” to halve the number of children affected by stunting by 2030, and the assessment of progress to date has not been possible for about a quarter of countries. Even fewer countries are expected to achieve the 2030 target of 3% prevalence for overweight, with just one in six countries currently “on track”. Furthermore, an assessment of progress toward the wasting target is not possible for nearly half of countries. Most low- and middle-income countries (LMICs) have used drops of capillary blood for the determination of hemoglobin concentration. This method is unreliable; thus, estimations of the prevalence of anemia and progress toward targets are likely inaccurate [4].

## 2. Challenges of Our Current Public Health Approach

**Figure 1:**
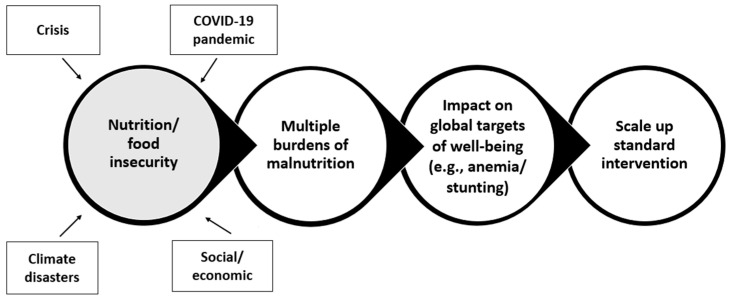
Linear logic approach to nutrition and food insecurity aspects of public health crises.

Currently, linear logic is used to address nutrition and food insecurity aspects of public health crises (Figure 1). This approach is understandably focused on a macro level to address public health programs and policy. Due to the focus primarily on the presence of a problem rather than its etiology or functional impact, and because it ignores the biological and environmental aspects of health and disease, this linear logic has limited ability to address specific diet, nutrition, and health issues.

In order to develop evidence-informed, safe, efficacious, and equitable programs and interventions to address nutrition and food insecurity issues while avoiding unintended consequences, we must be able to not only detect and assess the presence of a problem but also its etiologies. The following case study highlights the challenges and opportunities of a new approach to addressing the intersection of diet, nutrition, and health.

## 3. Case Study: What Is the “Double Burden”?

According to the World Health Organization (WHO), the “double burden” of malnutrition is characterized by the coexistence of undernutrition along with overweight and obesity or diet-related noncommunicable diseases within individuals, households, and populations and across the life course [5]. But even within this definition, how can we precisely understand the etiology and role of nutrition, genetics, health, and the environment? Moreover, as alluded to above, there are more than just two “burdens” to which malnutrition contributes. Indeed, there are several (ranging from wasting to obesity) physiological responses to illness and inflammation. So, from either a clinical or public health perspective, how can we improve the precision of interventions if we do not know whether an adverse functional outcome, such as increased susceptibility to disease, is the result of exposure (i.e., food insecurity) leading to undernutrition, combined with nutritional pathologies leading to specific nutrient deficits [6], or the result of overconsumption of calories via a poor-quality diet? In terms of public health surveillance, how can we understand the true magnitude of the problem and avoid “double counting” when we measure the prevalence of obesity and micronutrient insufficiency separately (as in most surveys) [7]? So, although “the double burden of malnutrition” has become part of the global health vernacular, we posit that “the multiple burdens” of malnutrition is more accurate terminology [8].

## 4. Relevant Definitions

The implications of the intersection of food insecurity, malnutrition, and the complex global health scenario (including climate and environmental changes) for child health are well documented. Recent reports by Wrottesley et al. [9] and Khan et al. [10] attest to the impact of undernutrition and the growing presence of the multiple burdens of coexisting undernutrition and overweight/obesity in school-aged children in LMICs. Moreover, an emerging body of evidence indicates that nutrition is important to the health and development of children across this age spectrum [11,12]. While it is clear that “coexisting malnutrition” is a problem, it is not at all clear what the implications of the problem might be to an individual child’s health and development or how best to assess it. The conundrum starts with the definition. Table 1 includes several potential manifestations of the “multiple burden” of malnutrition in children.

Importantly, the mere presence and measurement of the magnitude and population prevalence of an apparent problem is not sufficient to inform a specific intervention if the etiology is unknown [13,14]. For example, when does anthropometry tell us how or why a patient is above or below a given weight or length/height cutoff? Does the measurement of a given biomarker of nutrient status tell us how the status was achieved (e.g., was it the result of appetite/intake, a physiological response to inflammation, or a biochemical anomaly due to a nutrient–xenobiotic interaction)? Importantly, our ability to be more precise in diagnosis/attribution requires attention to an individual’s nutritional ecology [15]. 

The complexity of these scenarios is exemplified by the coexistence of obesity with either iron or vitamin D deficiency. Clearly, these comorbidities can exist in the same individual, but their mere presence is insufficient to inform a diagnosis or intervention. In the case of vitamin D deficiency in an obese individual, in addition to inadequate exposure, there may be myriad metabolic, endocrine, or developmental reasons that explain the deficiency [16,17]. In the case of iron deficiency, this cannot simply be viewed as a matter of exposure (i.e., insufficient nutrient intake and bioavailability). Rather, the diagnosis and intervention must be informed by an understanding of the complex nature of iron homeo-stasis, which is influenced by infection and inflammation. Berton and Gambero [18] highlight this complexity by emphasizing the role of hepcidin and the inflammatory process in our understanding of iron homeostasis and the assessment of iron status in obese children.

Clearly, when confronted with the multiple burdens of malnutrition, consideration of both the etiology and the ecology of a patient’s condition will improve the precision of both the diagnosis and effectiveness of the intervention.

Key questions raised by the concept of multiple burdens of malnutrition include the following:

- How to assess the needs of children and adolescents in the context of both health promotion and risk reduction.
- Whether there are “critical periods” of risk across the developmental spectrum of children. For example, where does wasting end and stunting begin; when does stunting become a risk for obesity; and what factors in the breadth of the child’s environment need to be considered in the context of these risks?
- What are the metabolic/physiologic implications of concomitant over- and undernutrition, and how might these implications intersect with other acute or chronic diseases (e.g., are they predisposing conditions or outcomes)?
- How can interventions be designed to target specific etiologies to prevent, resolve, or improve malnutrition?
- How to evaluate relevant health outcomes (e.g., infectious or noncommunicable disease risk, cognitive/behavioral development, etc.) and the role of environmental factors (e.g., home, community, climate, etc.) that influence both the need for and outcomes of programs designed to address the nutritional needs of children across the developmental spectrum.
- How to integrate the relevant components of the child’s nutritional ecology (e.g., biological, developmental, and environmental) into context-specific, equitable public health programs to address the needs of children and adolescents.

Developing an informed nutrition prescription in the absence of answers to these questions is challenging. As such, due to the need to assess the direct impact of nutritional interventions on individuals across the life span, as well as the need to better appreciate the intersection of food, nutrition, and health and their interactions with the environment, an ecological approach to assessment is needed.

## 5. Goals of Nutritional Assessment: How Can We Improve Precision?

What exactly do we mean by precision? Is it the ability to differentiate between health and disease in individuals and to compare the prevalence of a condition among populations or before and after an intervention? Or is it, more broadly speaking, the quality, condition, or fact of being sensitive or specific and precise (i.e., more inclusive of the sources of variability that might affect a given outcome of interest)? The former concerns methodology and the ability to correlate a response with an indicator; the latter depends on the ability to identify multiple interactions with the indicator. Both are critical. But to date, we have neither the ability to measure the multiple burdens of malnutrition nor the means to accurately assess the etiology of nutrition-related problems. 

This lack of valid, comprehensive, and feasible assessment techniques globally reduces the ability to implement effective and equitable nutrition interventions.

With respect to nutritional status and a given health outcome, our ability to understand the role of nutrition in health is driven by our capacity to address the following four fundamental questions:

1. Where do normal nutrient requirements end and specific health/physiological condition-related interactions and needs begin?
2. What is the role of diet/nutrition in conditions that require special consideration above and beyond the provision of a balanced diet that contains all essential nutrients required for growth, development, and health?
3. What is the role of factors within a child’s internal (health, genetics, developmental stage, etc.) and external (home, school, community, food system, physical) environment that contribute to these differences?
4. What are the best types and amounts of evidence to support the establishment of standards of care and the development of programs to address the role of nutrition in health promotion and disease prevention?

In addition to these fundamental considerations is the ability to address plausible metabolic mechanisms that may cause a deviation in the ability of normal nutritional status to prevent or attack parasites and infectious processes and/or affect predictors/causes of unintended consequences resulting from either clinical or public health interventions. 

Nutritional status is the operational measure of the adequacy of the diet to support health and is the result of a series of genetic, behavioral, physiological, and metabolic processes involved in acquiring and using the required dietary substances/nutrients to support the growth, repair, and maintenance of the body as a whole or in any of its parts [19]. Conceivably, from both a clinical and a public health perspective, nutritional assessment should be viewed as involving more than one nutrient and, functionally, as the potential interactions of multiple nutrients within biological systems of interest.

With specific regard to the application of an ecological approach to nutritional assessment, the components include exposure followed by nutrient status assessment and some measure of the health context, including nutrition-specific or nutrition-sensitive function. The core questions and clarifying sub-questions to be addressed in a comprehensive nutritional assessment include the following:

1. Are individuals/populations consuming nutrients at recommended levels, and if not, why?
2. Is there a health concern that is affecting appetite?
  - Is the individual/population food/nutrition insecure (i.e., are there access/availability issues)?
  - Is the individual/population adversely affected by “environmental” (climate, land/water resources, urban/rural/social/economic/political conditions) factors that are negatively affecting their food system and consequent food and nutrition security?
3. Are the processes of nutrition (i.e., the digestion, absorption, assimilation, and utilization of these nutrients)—which affect nutritional status—affected in individuals/populations due to the following:
  - Health context (infection, inflammation, NCDs, genetics (e.g., prevalent hemoglobinopathies), xenobiotics (therapeutic/recreational drugs, toxins))?
  - Severe prolonged stress due to crisis (e.g., war, displacement, refugee status, etc.) or climate stress (e.g., prolonged heat, drought, severe storms, etc.)?
4. Are there elements of an individual’s unique biology (life stage, health status, or environment) that affect either 1 or 2 above that should be measured?

**Figure 2:**
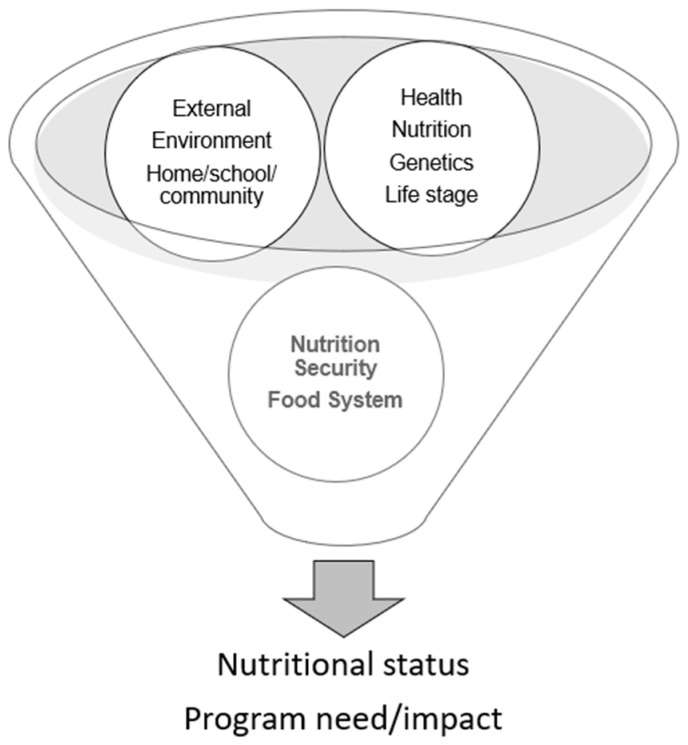
Ecological approach to nutritional assessment.

To be most impactful and avoid unintended consequences, the assessment of nutritional status should account for the role of nutrition as both a health input and outcome. Such an assessment acknowledges the complexity of nutrition and its biology as a complex biological variable with reciprocal relationships with both its internal and external environments (i.e., an ecology) [20]. Therefore, nutritional status can only be accurately assessed and interpreted by considering the health context, interactions with environmental factors, and other relevant aspects of an individual’s nutritional ecology (Figure 2). Consequently, assuming that we can answer questions about diet, nutrition, health, and environmental adaptation by limiting our focus to the first component (i.e., exposure/ingestion) of this complex biological process ignores the value of both the nutritional ecology and of an integrated “syndromic” approach to assessment.

## 6. Application of an Ecological Approach to Nutritional Assessment

To optimize overall well-being, we need to determine how best to assess the role of nutrition and its biology in a child’s health, and, more specifically, in conditions that might be associated with any of the multiple burdens of malnutrition described above. For example, what are the “burdens” and what role does nutrition play and why? 

The WHO Integrated Management of Childhood Illness (IMCI) is a well-established set of guidelines that employs an ecological approach to child health and clinical care [21]. The effectiveness of the IMCI platform in low-resource settings has been demonstrated [22,23], albeit in a limited fashion with specific regard to nutrition [24]. The IMCI strategy is generally applied to children <5 years old, but, in principle, it could be adapted and applied with a greater emphasis on nutrition to children > 5 years old. The key elements and objectives of the IMCI strategy are described in Table 2.

The IMCI platform implicitly describes an approach to the clinical assessment of children but does not provide an approach that could use the data generated to make public health policy (i.e., an approach to public health surveillance that could support public health interventions to address multiple forms of malnutrition and concomitant comorbidities). 

**Figure 3:**
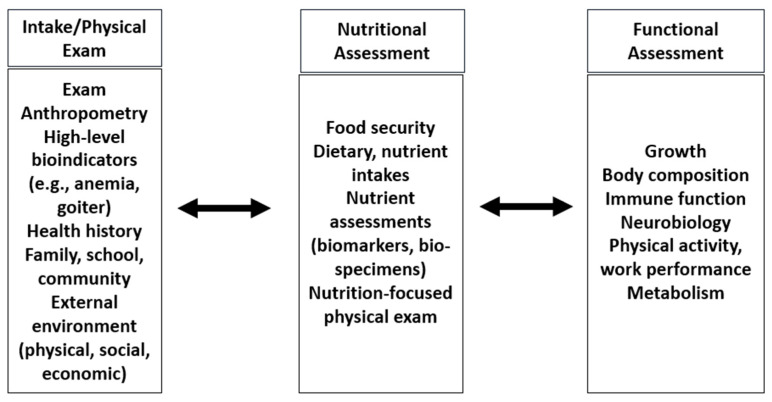
Application of an ecological approach to assessment.

An ecological approach to nutrition assessment can be used clinically and to aid in the development of surveys that capture the multiple burdens of malnutrition (Figure 3). Such a “syndromic” approach determines not only the presence but also the etiology of problems in order to inform intervention strategies.

**Figure 4:**
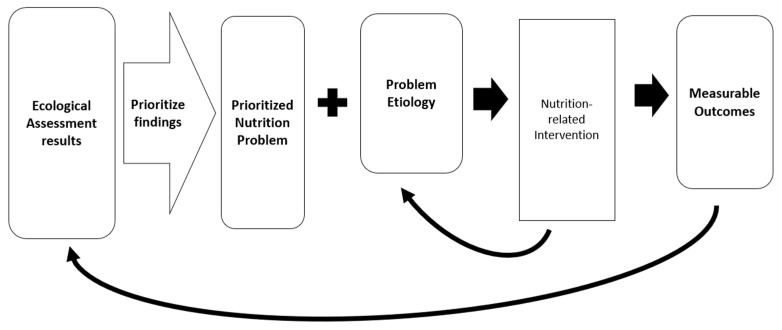
Assessment of findings and decisions to optimize outcomes.

Comprehensive nutritional assessments, which include the context impacting nutritional status, provide an opportunity to design an intervention that specifically targets the cause of the problem. To do this, the assessment findings must be prioritized, and a modifiable etiology identified. Modifiable etiologies are not always evident, but identifying one is important to design an intervention. However, in the absence of a cause that can be addressed with an intervention, the nutrition problem itself can sometimes be targeted, but it is always important to identify the biological mechanisms underlying the problem. Literature is emerging that demonstrates the effectiveness of targeting the etiology of a nutrition problem to increase the likelihood of resolution. For example, in a population of US Veterans, Lewis et al. [25] showed that a Nutrition Care Process significantly improved nutrition diagnoses and resolved nutrition problems. The flow of assessment findings and decisions that can be made to optimize outcomes is shown in Figure 4.

Importantly, this approach can be used across settings and populations. Implementation research focused on understanding the impact of the ecological approach can then be used to further develop the model. Furthermore, as nutrition-related assessment tools, biomarkers, and interventions are developed and validated, they can be integrated into the ecological model and tested in real-world scenarios to determine their impact on health outcomes.

### Challenges to Implementation

While the ecological model is a comprehensive and systematic way to think about assessment and the design of interventions, realities in the environment where these activities will occur must be considered. Examples of some of the structural/systemic challenges facing those attempting to implement new approaches to address priority nutritional targets have been recently highlighted [26,27]. Although the integration of key systems (i.e., agricultural, health, environment, and policy) will need to be created and reinforced, particularly in LMICs, once completed, a critical component in our ability to actualize new approaches to assessment and care such as those advocated in this paper will require increased commitment to developing the essential cadres of personnel to carry out such tasks. 

Within the United States and most LMICs, there is a dearth of trained nutrition care professionals, such as registered dietitians (RDs). As of 2021, there were approximately 104,000 RDs in the United States [28]. Currently, the United States has the highest number of RDs in the world, and still, it only has approximately 1 RD per 3160 people (based on RD data and US population estimates from 2021). In contrast, the ratio for physicians in the United States is closer to 1 physician per roughly 350 people (based on physician data and US population estimates from 2019) [29]. In countries with very few or no highly trained nutrition care professionals (e.g., RDs), comprehensive nutrition assessment in the community is extremely difficult or impossible. Thus, not only do we need to revolutionize how we conduct nutrition assessments, particularly for vulnerable populations, but we also need to address workforce capacity.

Workforce capacity for nutrition in many countries, both high and LMICs, is severely limited, and consequently, the interventions shown to be effective in research may never be fully implemented because the needed professionals are nonexistent. While many publications have addressed the lack of workforce, key strategic documents do not specifically cite nutrition professionals within their strategies. An example of this is the Global strategy on human resources for health: Workforce 2030 [30], which does not include nutrition or dietetic professionals. To address this issue, country-level nutrition plans and government agencies should recognize the importance of a nutrition workforce trained in comprehensive nutrition assessment and care. The allocation of resources for training nutrition professionals (e.g., dietitians and nutritionists), within the environment and from the populations they will be serving, is essential to meeting global goals related to the multiple burdens of malnutrition. 

## 7. Conclusions

We live in an ever more complicated global health context. This complexity demands a more comprehensive approach that addresses not just the presence and magnitude of a problem but, most importantly, its etiology. Coining terms like the “double burden” is deceptive both for describing and resolving the problem. Rather, nutrition is a biological variable that is intimately and inextricably involved in all aspects of human health and disease. As such, our ability to identify the specific etiologies of malnutrition and design interventions that address the complex intersection of food, nutrition, and health demands an ecological approach that recognizes this complexity and supports accurate assessment and attribution. Ultimately, this ecological approach will aid in the design and implementation of interventions to improve the health of those in greatest need.

## Figures and Tables

**Table 1:** Types of multiple burdens of malnutrition in children.

Coexisting Conditions	Example	Consideration
Low and excessive intake of nutrients	Multiple burdens of malnutrition, including the so-called “double” (over- and undernutrition) and “triple” burden (over-/under/micronutrient deficiencies	Implied nutrition insecurity, poor dietary diversity/overreliance on nutrient-limiting staple crops, etc. May be multifactorial: influenced by home, community, food system, physical environment.
Nutrition-sensitive condition and disease	Stunting and obesity	What is the nature of this relationship? Dietary, metabolic, behavioral, genetic, environmental?
Malnutrition and nutrient-related condition	Anemia and obesity	Obesity and anemia are both multifactorial conditions. Assessment and intervention must consider the role of biology, environment, and behavior.
Malnutrition and disease	Obesity and HIV, TB, COVID-19, etc.	What is the role of nutrition as an input (affecting susceptibility) or an outcome (affecting treatment/recovery/health status) of disease?
NCD and ID	MetSyn and HIV or TB	While not nutrition-specific, nutrition may play a role as both an input and an outcome that will affect susceptibility and response to treatment for these conditions.
ID and ID	HIV and TB	

Abbreviations: HIV, human immunodeficiency virus; ID, infectious disease; MetSyn, metabolic syndrome; NCD, noncommunicable disease; TB, tuberculosis.

**Table 2:** WHO Integrated Management of Childhood Illness (IMCI) strategy [21].

Components	Improves case management skills of HCPs
	Improves health systems to provide quality care
	Improves family and community health practices for health, growth, and development
In health facilities	Promotes the accurate identification of childhood illnesses in outpatient settings
	Ensures appropriate combined treatment of all major conditions that affect a young child
	Strengthens the counseling of caretakers and speeds up the referral of severely ill newborns and children
In the home	Promotes appropriate care-seeking behaviors
	Promotes improved nutrition and support for early childhood development
	Promotes illness prevention and correct implementation and adherence to treatment
Why IMCI is better than single-condition strategies	Considers each child in a holistic way
	Algorithms account for a variety of conditions that may place a newborn or child at risk of preventable mortality or impaired growth and development
	Focuses on effective case management and prevention of disease
	Contributes to healthy growth and development through immunization and nutritional and developmental counseling

HCPs, healthcare providers; IMCI, Integrated Management of Childhood Illness.
